# Measuring lung water adds prognostic value in heart failure patients undergoing cardiac magnetic resonance

**DOI:** 10.1038/s41598-021-99816-6

**Published:** 2021-10-11

**Authors:** Bruno M. L. Rocha, Gonçalo J. L. Cunha, Pedro Freitas, Pedro M. D. Lopes, Ana C. Santos, Sara Guerreiro, António Tralhão, António Ventosa, Maria J. Andrade, João Abecasis, Carlos Aguiar, Carla Saraiva, Miguel Mendes, António M. Ferreira

**Affiliations:** 1 Cardiology Department, Hospital de Santa Cruz, Centro Hospitalar Lisboa Ocidental, Av. Prof. Dr. Reinaldo dos Santos, 2790-134 Carnaxide, Lisbon Portugal; 2 Radiology Department, Centro Hospitalar Lisboa Ocidental, Av. Prof. Dr. Reinaldo dos Santos, 2790-134 Carnaxide, Lisbon Portugal

**Keywords:** Cardiology, Health care, Medical research

## Abstract

To assess whether a simplified cardiac magnetic resonance (CMR)–derived lung water density (LWD) quantification predicted major events in Heart Failure (HF). Single-centre retrospective study of consecutive HF patients with left ventricular ejection fraction (LVEF) < 50% who underwent CMR. All measurements were performed on HASTE sequences in a parasagittal plane at the right midclavicular line. LWD was determined by the lung-to-liver signal ratio multiplied by 0.7. A cohort of 102 controls was used to derive the LWD upper limit of normal (21.2%). The primary endpoint was a composite of time to all-cause death or HF hospitalization. Overall, 290 patients (mean age 64 ± 12 years) were included. LWD measurements took on average 35 ± 4 s, with good inter-observer reproducibility. LWD was increased in 65 (22.4%) patients, who were more symptomatic (NYHA ≥ III 29.2 vs. 1.8%; *p* = 0.017) and had higher NT-proBNP levels [1973 (IQR: 809–3766) vs. 802 (IQR: 355–2157 pg/mL); *p* < 0.001]. During a median follow-up of 21 months, 20 patients died and 40 had ≥ 1 HF hospitalization. In multivariate analysis, NYHA (III–IV vs. I–II; HR: 2.40; 95%-CI: 1.30–4.43; *p* = 0.005), LVEF (HR per 1%: 0.97; 95%-CI: 0.94–0.99; *p* = 0.031), serum creatinine (HR per 1 mg/dL: 2.51; 95%-CI: 1.36–4.61; *p* = 0.003) and LWD (HR per 1%: 1.07; 95%-CI: 1.02–1.12; *p* = 0.007) were independent predictors of the primary endpoint. These findings were mainly driven by an association between LWD and HF hospitalization (*p* = 0.026). A CMR-derived LWD quantification was independently associated with an increased HF hospitalization risk in HF patients with LVEF < 50%. LWD is a simple, reproducible and straightforward measurement, with prognostic value in HF.

## Introduction

Congestion is a central feature of Heart Failure (HF) often found in chronic stable disease^1^ and acute HF decompensation^2^. Pulmonary oedema plays a key role in several of the cardinal HF manifestations, namely dyspnoea and exercise intolerance. Albeit sensitive, these symptoms are not specific^3^ and, concurrently with left ventricular ejection fraction (LVEF) assessment, may influence crucial decisions in HF treatment (e.g., cardiac resynchronization therapy and/or implantable cardioverter defibrillator)^4^. Various fluid measuring methods have been proposed to accurately determine pulmonary extravascular water. Its quantification by thermodilution has been shown to correlate with increased left atrial, pulmonary wedge and/or diastolic pressures in series of patients with acute myocardial infarction^5^, chronic coronary syndrome^6^ and HF^7^. Imaging tests, such as chest radiography^8^ and lung ultrasonography^9^ are currently used in clinical practice as semi-quantitative measurements to assess the burden of pulmonary congestion and to discriminate it from non-cardiac causes of increased lung fluid. Whether routine evaluation, in addition to physical examination, may guide treatment in order to improve outcomes and whether these strategies may predict major events in the long-term (beyond 6 months) are yet to be thoroughly ascertained.

Lung Water quantification using Cardiac Magnetic Resonance (CMR) imaging has been shown to be feasible and is well correlated with the gold standard (*post-mortem* weighted gravimetric method)^10,11^. Added to its accuracy, CMR has the advantage of measuring lung water non-invasively and as a perfusion and ventilation-independent technique. Furthermore, it has been recently demonstrated that Lung Water Density (LWD) measured by this method independently predicts all-cause death, cardiovascular hospitalization or emergency department visit within 1-year in a cohort of patients with or at-risk of HF. However, the methodology proposed by Thompson et al.^12^ may be demanding and time-consuming, rendering CMR-derived LWD a less useful tool in everyday clinical practice. Thus, we aimed to assess whether a simplified CMR-derived LWD determination was feasible and correlated with major events in patients with HF and LVEF < 50%.

## Methods

### Study population

Consecutive patients with HF referred to CMR imaging were screened and those with a LVEF < 50% at CMR and follow-up in our centre from 2016 to 2018 were included. Patients aged < 18 years and those with known chronic lung disease and/or chronic liver disease (as determined by their physician) were excluded. Patients without available Half-Fourier Acquisition Single-shot Turbo spin Echo imaging (HASTE) sequences (n = 4), and those whose HASTE images had significant contribution from the heart, large blood vessels and/or hilar structures (n = 4) were also excluded. The study protocol was reviewed and approved by the local ethics' committee—Comissão de Ética para a Saúde do Centro Hospitalar de Lisboa Ocidental, with the Registry number 20170700050—, which waived the need for informed consent. This investigation was performed in accordance with the Declaration of Helsinki.

### Demographic, clinical and laboratory data

Demographic, clinical and laboratory data were retrospectively collected from the patient chart and electronic medical records (within a 6-month window previous to CMR). HF diagnosis was defined according to the 2017 ACC/AHA/HFSA Guidelines^13^.

### CMR data acquisition and lung water analysis

All subjects were imaged using a 1.5 T scanner (Siemens Avanto®, Siemens Healthineers, Erlangen, Germany). Cardiac function and structure were evaluated by using a balanced steady-state free precession cine sequence with retrospective ECG-gating. Ventricular volumes were measured by experienced Cardiologists and Radiologists using a dedicated software (Circle Cardiovascular Imaging® release 5.6.4, Calgary, Canada).

**Figure 1:**
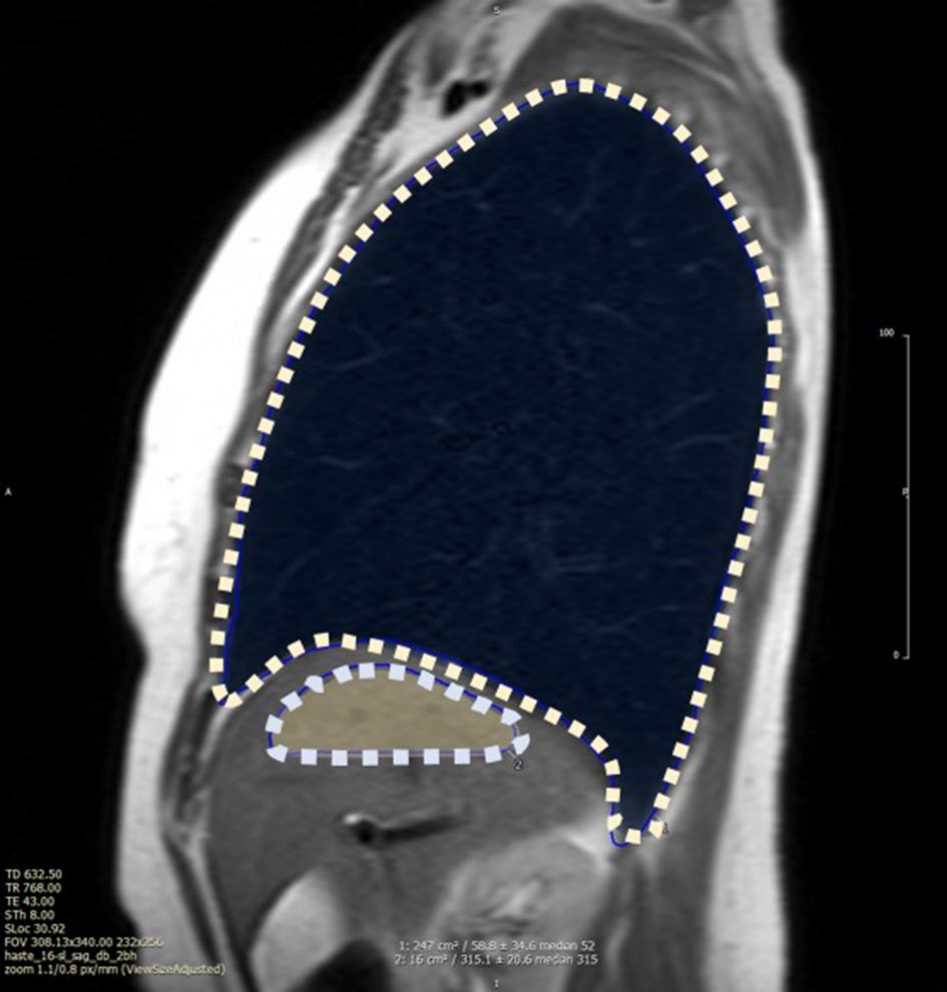
Simplified method for imaging Lung Water: Lung and Hepatic operator-selected region of interest (ROI) are outlined in the parasagittal plane at the larger cross-sectional lung area, usually at the right midclavicular line. Lung water density is calculated by the lung-to-liver signal ratio multiplied by 0.7.

Lung water was measured using a HASTE pulse sequence. Typical imaging parameters included a field of view of 340 × 340 mm, 8 mm slice thickness, 5/8ths partial Fourier, 780 Hz/pixel bandwidth and a 120°–180° refocusing pulse flip angle and ECG-gated image acquisition during diastasis. A single sagittal HASTE slice at the right midclavicular line at end-expiratory breath-hold was used to measure lung and liver signal intensities. Two regions of interest (ROI) were manually drawn: one including all visible right lung tissue (excluding pleural effusion and any heart volume, when present), and another including the upper half of the liver. Finally, LWD (%) was determined as the lung-to-liver signal ratio multiplied by 0.7^14^—Fig. 1. LWD measurements were performed by two independent observers (BR, GC) blinded to all patient data.

Briefly, the major differences between this simplified method for LWD measurement and the previously reported one^12^ are: (1) lack of 30 min supine positioning before CMR; (2) single sagittal HASTE sequence acquired at end-expiratory breath-hold versus 20 repeats during free-breathing with subsequent retrospective selection of end-expiration images.

### Control cohort

A retrospective cohort of patients undergoing CMR imaging for supraventricular or ventricular premature beats on 24-h Holter monitoring, doubtful structural heart disease on echocardiography and those prior to paroxysmal atrial fibrillation (AF) ablation were screened. Those without known cardiovascular risk factors other than age and sex, no demonstrable structural heart disease nor late gadolinium enhancement were included as controls (n = 102). This cohort was used to derive the upper limit of normal [i.e., two standard deviations (SD) above the mean] of the LWD (21.2%).

### Follow-up and endpoint definitions

Hospitalization for acute HF was defined as per the ACC/AHA/HFSA Guidelines^13^. Patients were assessed at least annually and data on clinical status and HF-related events were documented in the electronic medical records. The primary endpoint was a composite of time to all-cause death or HF hospitalization. Three Cardiologists (BR, GC, PF) performed event adjudication and any disagreements were discussed within the panel and resolved by consensus.

### Statistical analysis

Categorical values are presented as counts (and percentage) and continuous variables as mean ± SD (normal distribution) or median [interquartile range (IQR)] (nonparametric). Pearson’s Chi-squared (χ^2^) test, Mann–Whitney U and independent samples t-test were applied for comparison where appropriate. Bland–Altman analysis and Lin’s concordance correlation coefficient were used to evaluate the inter-observer agreement between LWD measurements as a continuous variable. Cohen’s κ was used to assess inter-observer reliability in categorizing LWD as increased.

Univariate analysis was applied to relate a broad range of clinical and CMR parameters to the study endpoint. After multicollinearity correction, variables with a *p*-value < 0.05 were then selected for multivariate analysis. LWD was imputed into the models as either a continuous variable or reclassified as the percentage (per 1%) above the upper limit of normal when it was increased (> 21.2%) and as a null value whenever within normal range (0 to 21.2%). The CMR time point served as the index time for time-to-event analyses which were performed using Cox-regression hazards model and Kaplan–Meier survival curves. Multivariate competing risk analyses of predictors for time to HF hospitalization was performed with the Fine and Gray proportional subdistribution hazard regression model. Subgroup analysis included: (1) HF with reduced LVEF (< 40%) vs. midrange LVEF (40–49%); (2) asymptomatic or mild HF symptoms [New York Heart Association (NYHA) I–II] vs. moderate or severe HF symptoms (NYHA III–IV); (3) increased NT-proBNP (> 600 or 900 pg/mL if AF) vs. “normal” NT-proBNP (≤ 600 or 900 pg/mL if AF); and (4) status at CMR acquisition (during HF hospitalization vs. outpatient).

A prespecified multivariate model adjusted for age and NT-proBNP was constructed with events right-censored at 1-year follow-up in order to ascertain whether results were similar to those previously reported in the literature^12^.

Statistical analysis was performed using SPSS v26.0 and STATA v13. Statistical significance was set at *p*-value < 0.05 (two-sided).

### Ethics approval and consent to participate

The hospital ethics’ committee approved of this study at 07th September of 2020 (RNEC 20170700050). The requirement of informed consent to participate and publication was waived, based on the Portuguese Deliberation n.º 1704/2015 of the National Commission for the Protection of Data (CNPD) and the Legislation under the Laws n.º 58 and 59/2019, n.º 12/2005 and the Data Protection General Regulation (UE) 2016/679.

## Results

### Cohort demographics and clinical data

The overall cohort included 290 HF patients with a mean age of 64 ± 12 years, most of whom were male (74.8%). HF aetiology was mainly ischaemic (56.2%) and mean LVEF was 34 ± 10%. Baseline demographics are depicted in Table 1. CMR was acquired during hospitalization in 69 patients (23.7%).

**Table 1:** Baseline demographics, CMR measurements and hard outcomes in patients with “dry lungs” (LWD ≤ 21.2%) and “wet lungs” (LWD > 21.2%).

	Normal LWD “Dry Lungs” (n = 225)	Increased LWD “Wet Lungs” (n = 65)	*p*-value
Age, mean ± SD (years)	64.2 ± 12.5	62.5 ± 10.3	0.321
Male sex, n (%)	173 (76.9%)	44 (67.7%)	0.132
Hypertension, n (%)	157 (69.8%)	39 (60.0%)	0.138
Diabetes mellitus, n (%)	50 (22.2%)	21 (32.3%)	0.096
Atrial fibrillation, n (%)	55 (24.4%)	24 (36.9%)	0.047
MDRD, mean ± SD (mL/min/1.73 m^2^)	78.7 ± 27.4	71.7 ± 25.4	0.069
Ischaemic aetiology, n (%)	126 (56.0%)	37 (56.9%)	0.895
Dilated cardiomyopathy, n (%)	64 (28.4%)	22 (33.8%)	0.401
NYHA I, n (%)	97 (43.1%)	20 (30.8%)	0.017
NYHA II, n (%)	94 (41.8%)	26 (40.0%)	
NYHA III, n (%)	29 (12.9%)	13 (20.0%)	
NYHA IV, n (%)	4 (1.8%)	6 (9.2%)	
NT-proBNP, median (IQR) (pg/mL)	802 (355–2157)	1973 (809–3766)	< 0.001
Beta-blockers, n (%)	181 (80.4%)	57 (87.7%)	0.180
ACEi, n (%)	163 (72.4%)	41 (63.1%)	0.145
ARB, n (%)	42 (18.7%)	10 (15.4%)	0.543
ARNi, n (%)	9 (4%)	4 (6.2%)	0.460
MRA, n (%)	83 (36.9%)	35 (53.8%)	0.014
Ivabradine	9 (4%)	4 (6.2%)	0.460
Furosemide, n (%)	95 (42.2%)	34 (52.3%)	0.150
Furosemide dose, median (IQR) (mg/day)	0 (0–40)	20 (0–40)	0.112
CMR during hospitalization, n (%)	45 (20.0%)	24 (36.9%)	0.005
LVEDVi, mean ± SD (mL/m^2^)	113.5 ± 38.1	139.5 ± 48.2	< 0.001
LVEF, mean ± SD (%)	34.8 ± 8.8	29.3 ± 10.6	< 0.001
LWD at right lung, median (IQR) (%)	14.8 (12.5–17.8)	26.3 (22.7–30.5)	< 0.001
Death or HF hospitalization, n (%)	29 (12.9%)	25 (38.5%)	< 0.001
All-cause death, n (%)	17 (7.6%)	3 (4.6%)	0.410
HF hospitalization, n (%)	16 (7.8%)	24 (37.5%)	< 0.001

*ACEi* angiotensin-converting enzyme inhibitor, *ARB* angiotensin II receptor blocker, *ARNi* angiotensin receptor-neprilysin inhibitor, *CMR* cardiac magnetic resonance, *HF* heart failure, *IQR* interquartile range, *LV* left ventricle, *LVEDVi* left ventricle end-diastolic volume index, *LVEF* left ventricular ejection fraction, *LWD* lung water density (%), *MDRD* modification of diet renal disease, *MRA* mineralocorticoid receptor antagonist, *NYHA* New York Heart Association, *SD* standard deviation. Clinical and laboratory data was collected from electronic medical records whenever available within a timeframe of 6 months.

### LWD analysis

ROI tracing for LWD measurements lasted on average 35 ± 4 s. The simplified LWD quantification method was associated with good reproducibility: Lin’s concordance correlation coefficient of 0.95 [95% Confidence Interval (CI) 0.935–0.965; *p* < 0.001], with minimal bias according to the Bland–Altman analysis (bias 0.17%; 95% limits of agreement: -3.6% to 3.9%)—Fig. S1. Likewise, a good agreement (Cohen’s κ = 0.78; *p* < 0.001) was found between the two raters for the categorization of LWD as elevated.

LWD histogram is illustrated in Fig. S2. Increased LWD (> 21.2%) was observed in 65 (22.4%) patients. Even though this group of patients had a higher median NT-proBNP and a lower LVEF (Table 1 and Fig. S3), correlations between LWD and NT-proBNP or LVEF were weak (Spearman rho 0.26 and − 0.25, respectively—Fig. S3). Additionally, the percentage of patients with increased LWD—“wet lungs”—increased with worsening NYHA class (Fig. S4). Nevertheless, the number of patients with normal LWD—“dry lungs”—remains considerably high (> 30%) across the spectrums of NT-proBNP, LVEF and NYHA class. Overall, LWD appears to add incremental information that is relatively independent from NT-proBNP, LVEF and NYHA.

### Primary composite endpoint

During a median follow-up of 21 (IQR 13–29) months, 20 patients (6.9%) died and 40 (13.8%) had one or more admissions for HF. Six of the patients who died were previously hospitalized for HF.

**Figure 2:**
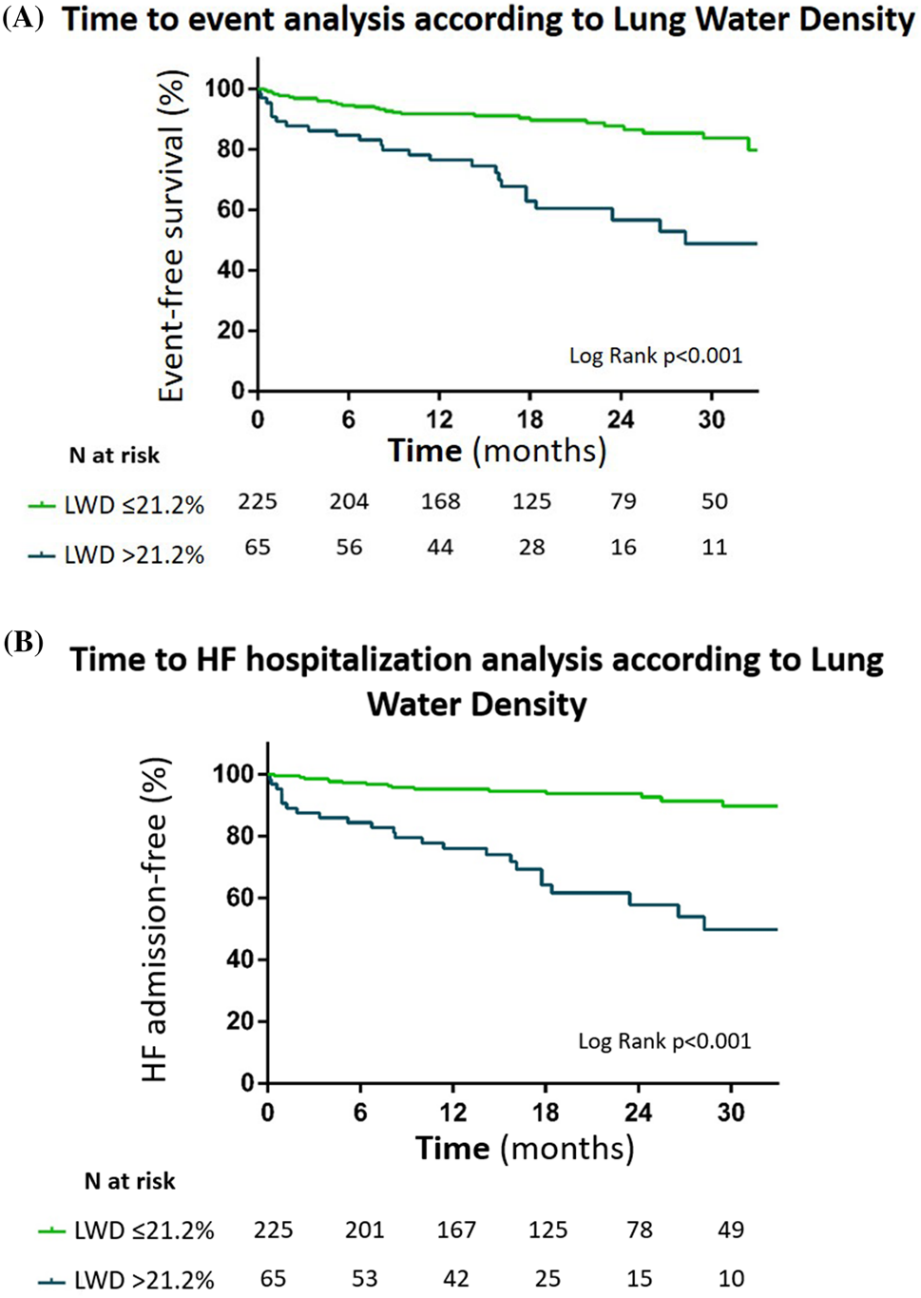
(**A**) Kaplan–Meier curves for 290 HF patients and LVEF < 50% with increased (> 21.2%) or normal LWD (≤ 21.2%) for the primary composite endpoint (i.e., all-cause death or HF hospitalization) presented as event-free survival (%) at 30 months. Compared to normal LWD, those with increased LWD were significantly more likely to have an event (log rank *p* < 0.001). *LWD* lung water density. (**B**) Kaplan–Meier curves for 290 HF patients and LVEF < 50% with increased (> 21.2%) or normal LWD (≤ 21.2%) for HF hospitalization presented right-censored at 30 months. Compared to normal LWD, patients with increased LWD were significantly more likely to have at least one HF hospitalization (log rank *p* < 0.001). *LWD* lung water density.

Compared to patients with normal LWD, those with increased LWD met more often the primary composite endpoint [25 (38.5%) vs. 29 (12.9%); *p* < 0.001], mostly due to HF hospitalization [24 (37.5%) vs. 16 (7.8%); *p* < 0.001). Univariate analyses to predict the primary endpoint are presented in Table 2. In the multivariate model (Table 3), LWD [Hazard ratio (HR) per 1%: 1.066; 95% CI: 1.018–1.115; *p* = 0.007] remained an independent predictor of the primary composite endpoint after adjusting for NT-proBNP, NYHA functional class, LVEF and serum creatinine. These findings were mainly driven by an association between increased LWD and time to first HF hospitalization (HR per 1%: 1.063; 95% CI: 1.007–1.122; *p* = 0.026), as adjusted for competing risks (Table S1). Event-free survival and time to HF hospitalization are depicted in Fig. 2.

**Table 2:** Univariate Cox regression model for the primary composite endpoint.

Variables	Univariate analysis		
	HR	95% CI	*p*-value
Male gender	0.940	0.500–1.757	0.850
Age (years)	1.020	1.000–1.046	0.070
Hypertension	1.050	0.600–1.851	0.860
Diabetes mellitus	0.520	0.300–0.909	0.020
BMI (Kg)	0.960	0.900–1.027	0.240
Atrial fibrillation	0.719	0.433–1.195	0.203
Previous MI	0.820	0.480–1.413	0.480
NT-proBNP (pg/mL), per unit	1.006	1.004–1.009	< 0.001
Serum creatinine, per 1 mg/dL	2.250	1.560–3.235	< 0.001
ACEi	0.920	0.500–1.691	0.790
ARB	1.810	0.770–4.225	0.170
MRA	0.690	0.400–1.177	0.170
ARNi	0.290	0.100–0.816	0.020
Beta-blocker	0.930	0.450–1.905	0.840
Ivabradine	0.410	0.160–1.025	0.060
Digoxin	0.860	0.270–2.764	0.800
Furosemide	0.310	0.170–0.543	< 0.001
NYHA class II	0.090	0.040–0.237	< 0.001
NYHA class III	0.180	0.080–0.431	< 0.001
NYHA class IV	0.460	0.190–1.109	0.080
LVEF, per 1%	0.940	0.910–0.964	< 0.001
LVEDVi	1.010	1.000–1.016	< 0.001
LWD, per 1%	1.090	1.051–1.130	< 0.001

*ACEi* angiotensin-converting enzyme inhibitor, *ARB* angiotensin II receptor blocker, *ARNi* angiotensin receptor-neprilysin inhibitor, *BMI* body mass index, *CI* confidence interval, *HR* hazard ratio, *LVEDVi* left ventricle end-diastolic volume index, *LVEF* left ventricular ejection fraction, *LWD* lung water density (%), *MI* myocardial infarction, *MRA* mineralocorticoid receptor antagonist, *NYHA* New York Heart Association.

**Table 3:** Univariate and multivariate Cox regression model for the primary composite endpoint.

Variables	Univariate analysis			Multivariate analyses		
	HR	95% CI	*p*-value	HR	95% CI	*p*-value
NYHA functional class^a^	1.923	1.100–3.333	0.020	2.398	1.300–4.425	0.005
NT-proBNP, per 100 pg/mL	1.006	1.004–1.009	< 0.001	1.000	0.996–1.004	0.954
Serum creatinine, per 1 mg/dL	2.250	1.560–3.235	< 0.001	2.507	1.364–4.609	0.003
LVEF, per 1%	0.940	0.910–0.964	< 0.001	0.966	0.935–0.997	0.031
LWD, per 1%	1.094	1.056–1.134	< 0.001	1.066	1.018–1.115	0.007

All variables (except NYHA functional class) were assessed as continuous variables.

^a^NYHA III–IV versus I–II; *CI* confidence interval, *HR* hazard ratio, *LVEF* left ventricular ejection fraction, *LWD* lung water density (%).

### Subgroup and sensitivity analyses

Multivariate analysis remained similar when evaluating LWD without imputing null values to LWD within the normal range (HR per 1%: 1.054; 95% CI: 1.018–1.092; *p* = 0.003) and when censoring follow-up at 1-year (HR for LWD > 21.2%: 2.851; 95% CI: 1.354–6.001; *p* = 0.006; HR per 1% LWD: 1.066; 95% CI: 1.026–1.109; *p* = 0.001).

**Figure 3:**
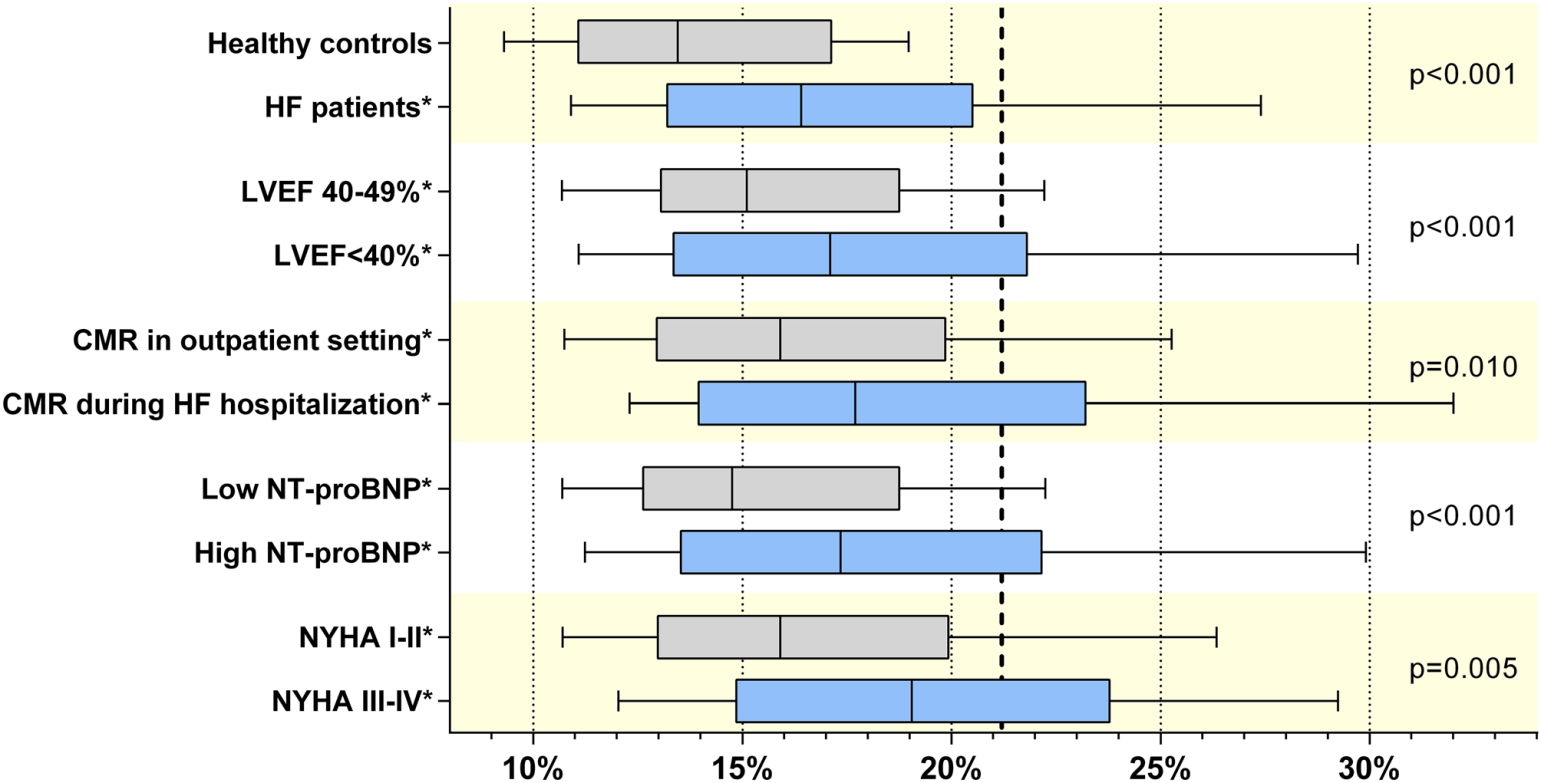
Distribution of LWD as per the following subgroups: (1) LVEF [reduced (< 40%) vs. midrange (40–49%) LVEF]; (2) functional class (NYHA I–II vs. III–IV); (3) NT-proBNP (> 600 or 900 pg/mL if AF vs. ≤ 600 or 900 pg/mL if AF); and (4) status at CMR acquisition (during HF hospitalization vs. outpatient); Box plots illustrating LWD median, 25th and 75th percentiles, and whiskers show the 10th and 90th percentiles; *p < 0.05 in comparison to control; *p*-values for LWD comparison between subgroups are shown in figure. *CMR* cardiac magnetic resonance, *HF* heart failure, *LVEF* left ventricular ejection fraction, *NYHA* New York Heart Association.

**Figure 4:**
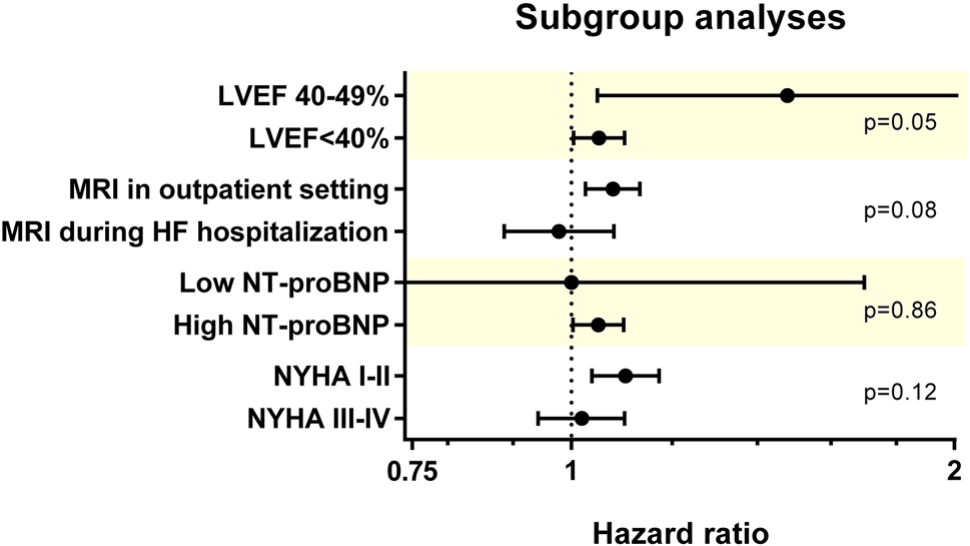
Primary composite endpoint analysis truncated at 1-year as per the following subgroups: (1) LVEF [reduced (< 40%) vs. midrange (40–49%) LVEF]; (2) functional class (NYHA I–II vs. III–IV); (3) NT-proBNP (> 600 or 900 pg/mL if AF vs. ≤ 600 or 900 pg/mL if AF, which is illustrated above as high vs. low NT-proBNP, respectively); and (4) and status at CMR acquisition (during HF hospitalization vs. outpatient). The HR (and 95% CI) of increased LWD (> 21.2%) for the primary endpoint is illustrated, adjusted to the variables used in the multivariate model (LVEF, serum creatinine, NT-proBNP and NYHA class). The results are consistent across different subgroups, particularly powerful in patients with LVEF 40–49% or whose MRI was performed in the outpatient setting. *CMR* cardiac magnetic resonance, *LVEF* left ventricular ejection fraction, *NYHA* New York Heart Association.

LWD distribution throughout the subgroups showed increased lung water in patients with reduced LVEF (< 40%), NYHA III–IV, increased NT-proBNP and CMR during HF hospitalization (Fig. 3). Repeated subgroup multivariate analysis demonstrated consistent and similar results to the overall population (Fig. 4).

## Discussion

We report the application of a simplified CMR-derived lung water quantification method in a cohort of patients with HF and LVEF < 50%. The major findings were as follows: (1) LWD is rapidly measurable in routine CMR without any additional technical procedure or incremental costs, with good inter-observer reproducibility and agreement; (2) the cut-off for “normal” LWD in our control population was similar to that reported in the study by Thompson et al. (20.8% vs. 21.2%)^12^; and (3) the simplified LWD measurement independently associated with an increased risk of the primary composite endpoint, with an early separation of the event-free survival curves, mainly due to an association between LWD and time to first HF hospitalization.

Persistent and recurrent congestion is associated with worse prognosis in HF^15–17^. Measuring lung water allows the detection and quantification of lung fluid, showing promise as both a prognostic marker and a potential therapeutic target to tailor diuretic treatment. Several methods have been proposed in clinical practice to extend and refine physical examination findings. Thermodilution is an accurate method compared to gold standard and, in addition, allows indirect measurement of lung injury. However, the technique is cumbersome and not widely available outside the critical care unit or the hemodynamic laboratory^18,19^. Similarly, tomographic bioimpedance-derived methods allow the accurate measurement of extravascular lung fluid^20,21^, but did not gain widespread use. In contrast, lung ultrasound, a method that allows water semi-quantification by assessing B-lines^22,23^, is being increasingly integrated into clinical practice in order to achieve euvolaemia in acute scenarios^24^. Moreover, ultrasound-guided diuretic treatment may facilitate symptom improvement and reduce the number of decompensations in chronic HF^9^. In comparison to other methods, LWD measured by CMR may allow a notably accurate and highly reproducible quantification of lung fluid that may be conceivably useful as a surrogate endpoint in studies focusing on strategies to tackle pulmonary congestion^25^.

Lung water content determination by CMR has been the focus of research for almost four decades^10^. Water was shown to be reliably measured by this method firstly in sponge phantoms, and later applied to lung measurements in animal models and humans^26,27^, and were correlated to invasively measured left-sided filling pressures in the latter^12^. Moreover, CMR has the advantage of accounting for topographical inhomogeneity in tissue water content^28^ that may go undetected by other means. Previously, Thompson et al. have investigated the prognostic role of LWD in patients with (n = 121) or at risk of HF (n = 82). Firstly, the derived upper limit of normal cut-off in the cohort was similar to the observed in our study (20.8% vs. 21.2%) which strengthens the clinical applicability of the simplified method. Secondly, increased LWD (> 20.8%) was an independent predictor of all-cause death, cardiovascular hospitalization or emergency department visit within 1-year in their cohort of patients with or at risk of HF and any LVEF^12^. In our study, we found similar results in a considerably larger cohort (n = 290) of patients with established HF and LVEF < 50% in whom LWD independently predicted all-cause death or HF hospitalization at a median follow-up of 21 months (HR per 1%: 1.07; *p* = 0.016). Thus, we further validate the prognostic value of a CMR-derived LWD determination in this population.

Thompson et al. were first to report the application of a CMR method for LWD estimation in a clinical population with established or at-risk of HF^12^. In the abbreviated protocol, measurements were performed in a single sagittal slice in the right lung at its largest cross-sectional area during free-breathing 20 repeats, separated for > 5 s (total scan time of about 2 min). Images were obtained approximately 30 min after supine positioning at the onset of the CMR exam^12^. In our study, we used routine HASTE images without any additional methodology concerns. Indeed, our study is retrospective in its design and CMR images were acquired without the specific purpose of measuring LWD. Despite this, images were appropriate for lung ROI outlining in all but four patients since slices had a significant contribution from the heart and/or hilar structures. Thus, not only does our investigation validates and strengthens the prognostic value of CMR-derived LWD determination in HF patients with LVEF < 50% but also simplifies the acquisition method. Notably, our findings suggest that CMR can be performed in the usual fashion regardless of LWD determination, allowing an accurate lung water quantification that has important prognostic value. Furthermore, lung and liver ROI outlining is a simple trainable task and an automated software tool may be easily applied.

Interestingly, we found that NT-proBNP, a strong established major outcome predictor in HF^29,30^, was not predictive of the primary endpoint in multivariate analysis once LWD was imputed into the model. Indeed, natriuretic peptides levels correlate with congestive symptoms and signs^31^, and, thus, LWD may represent a more refined measure of hypervolaemia. Nonetheless, the interpretation of these results may be complicated by the inclusion of patients whose CMR was performed during hospitalization, particularly as we collected NT-proBNP at the closest day available to the CMR and, while outpatient, we accepted a timeframe of 6 months.

We found that LWD was a strong predictor of meaningful events in HF. Of note, event-free survival curves diverged early, demonstrating LWD as a strong and early predictor of time to first HF hospitalization. Altogether, our investigation supports the systematic opportunistic measurement of LWD in HF with LVEF < 50% in order to further stratify patient risk. Whether LWD-targeted interventions are beneficial and whether LWD adds prognostic value to known and well-validated risk scores in HF, are interesting hypothesis worth being further investigated. Indeed, it would be appealing to test the predictive power of a CMR-HF score integrating LWD compared to the recommended risk score calculators. Finally, the targeted population in whom LWD measurement might be most useful (e.g. "subclinical congestion" detection at discharge or congestion in the outpatient setting) should be further investigated, as should one assess the clinical utility and cost-effectiveness of this method compared to others.

## Limitations

Some limitations should be acknowledged. First, we assumed that the liver CMR signal corresponds to a water density of 70% as formerly reported^32^. Despite having excluded patients with known chronic liver disease, the presence of either congestion or fatty liver infiltration and/or subclinical liver disease may have led to LWD underestimation or overestimation. Similarly, albeit excluding those who had known chronic lung disease, pulmonary function tests and chest-computed tomography were not systematically available, hence variables other than lung congestion may have led to lung density miscalculations. Second, regional variations and positional redistribution in lung water content were not accounted for, and whether other parameters, such as maximal LWD, are of higher prognostic value, were not ascertained. Furthermore, we could not determine the therapeutic effect of diuretic treatment given the retrospective nature of our study and given that most patients had “normal” LWD at the time of CMR. Whether lung congestion detected by CMR should be a therapeutic target (e.g. diuretic adjustments) is a hypothesis worth exploring prospectively. Although patients with acute HF most often had increased LWD compared to those in the outpatient setting, subgroup analysis were consistent with the main findings. Moreover, we included patients who were referred to CMR (selection bias) as determined by their attending physician and whether findings can be extrapolated to the overall HF population is debatable. Limitations inherent to a retrospective single-centre study design are to be recognized. Finally, the control group, while not having overt structural heart disease, was not a healthy cohort and the true normal cut-off of LWD might even be lower than that considered here. Nonetheless, the prognostic value of LWD was confirmed in sensitivity analysis where all values were including, regardless of cut-off.

## Conclusion

Lung water quantification by a simplified CMR-derived method independently associates with an increased risk of HF hospitalization in patients with HF with LVEF < 50%. LWD measurement is a simple, reproducible and straightforward method, further adding to the key prognostic role of CMR in HF. Future studies should prospectively assess the prognostic performance of LWD in comparison to and/or on top of known HF prognostic variables and well-validated risk scores.

## Supplementary Information

Supplementary Information 1.

## Data Availability

The datasets used and/or analysed for the current study are available from the corresponding author on reasonable request.

## Abbreviations

ACEi: Angiotensin-converting enzyme inhibitor
AF: Atrial fibrillation
ARB: Angiotensin II receptor blocker
ARNi: Angiotensin receptor-neprilysin inhibitor
BMI: Body mass index
CI: Confidence interval
CMR: Cardiac magnetic resonance
HASTE: Half-Fourier acquisition single-shot turbo spin echo imaging
HF: Heart failure
HR: Hazard ratio
IQR: Interquartile range
LVEDVi: Left ventricle end-diastolic volume index
LVEF: Left ventricular ejection fraction
LWD: Lung water density (%)
MDRD: Modification of diet renal disease
MI: Myocardial infarction
MRA: Mineralocorticoid receptor antagonist
NYHA: New York Heart Association
ROI: Region of interest
SD: Standard deviation
