# Supplementary material for: Measuring lung water adds prognostic value in heart failure patients undergoing cardiac magnetic resonance
Source: Sci Rep. 2021 Oct 11;11:20162. doi: 10.1038/s41598-021-99816-6 (PMC8505633; doi:10.1038/s41598-021-99816-6)
Supplement: Supplementary file 1 — Supplementary Information 1. [file 41598_2021_99816_MOESM1_ESM.docx]

**[TITLE]:** Measuring Lung Water adds prognostic value in Heart Failure patients undergoing Cardiac Magnetic Resonance

**[SHORT TITLE]**: Lung Water quantification by Cardiac MRI in HF

**[Authors]:** Bruno M.L. Rocha^1^, Gonçalo J.L. Cunha^1^, Pedro Freitas^1^, Pedro M.D. Lopes^1^, Ana C. Santos^2^, Sara Guerreiro^1^, António Tralhão^1^, António Ventosa^1^, Maria J. Andrade^1^, João Abecasis^1^, Carlos Aguiar^1^, Carla Saraiva^2^, Miguel Mendes^1^, António M. Ferreira^1^

[**Institutional Affiliations**]: ^1^: Cardiology Department, Hospital de Santa Cruz, Centro Hospitalar Lisboa Ocidental, Lisbon, Portugal; ^2^: Radiology Department, Centro Hospitalar Lisboa Ocidental, Lisbon, Portugal.

**[Address]:** ^1,2^Av. Prof. Dr. Reinaldo dos Santos, 2790-134 Carnaxide, Lisbon, Portugal

**[Corresponding Author] [Proofs]**

[Name]: Bruno M.L. Rocha

[Telephone]: +351 21 043 1000 | [Fax]: +351 21 043 15 89

[E-mail]: [bruno.rocha@campus.ul.pt](mailto:bruno.rocha@campus.ul.pt)

**Supplementary Figure 1** – (a) Bland-Altman analysis revealed minimal bias between the two raters; (b) Lin's concordance correlation coefficient (ρ_c_) was also associated with a very good agreement between raters. UL: upper limit; LL: lower limit.
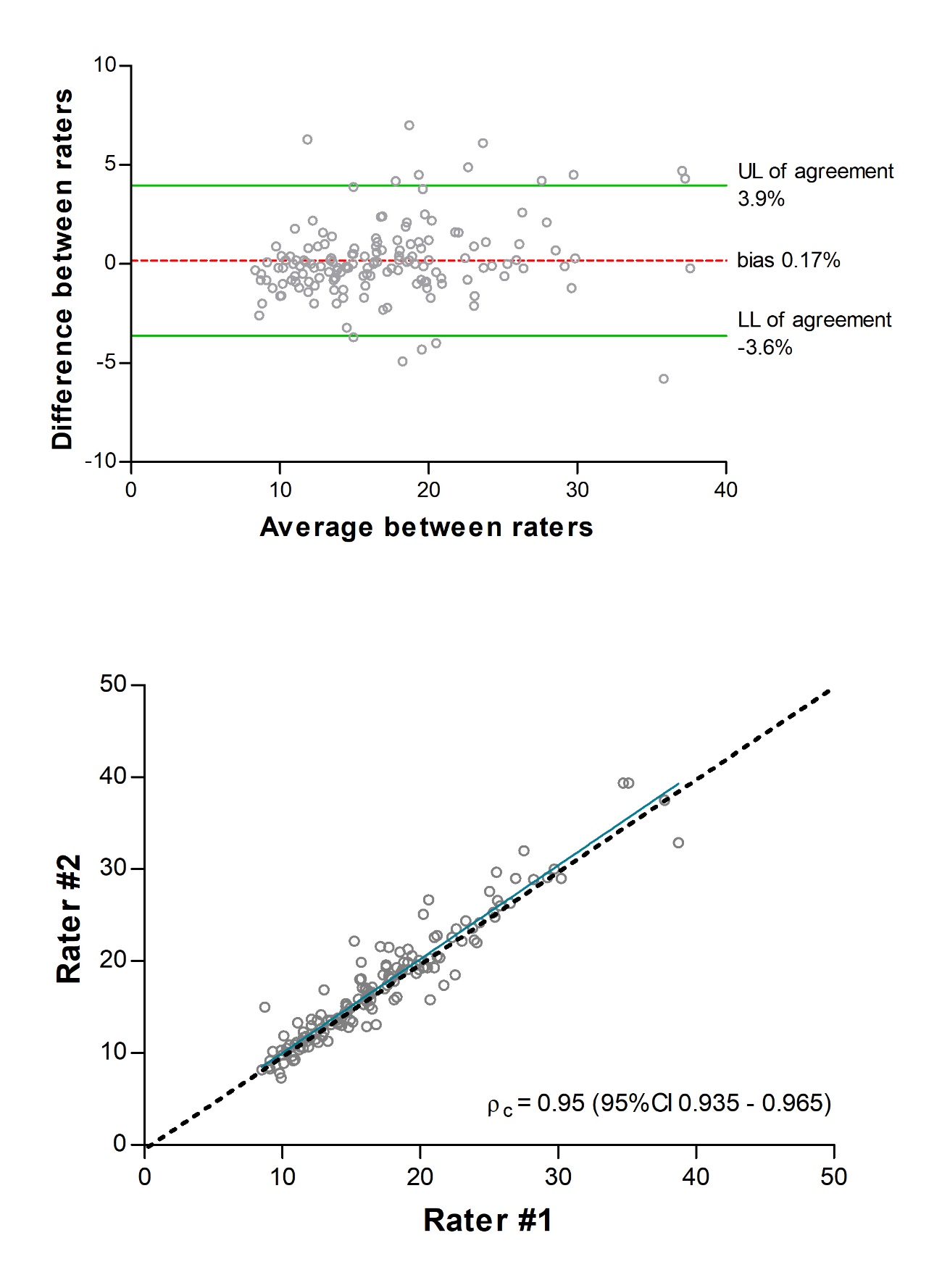


**Supplementary Figure 2** – Histogram showing the LWD distribution across the HF cohort; LWD >21.2% defined patients with “Wet” lungs, while those with LWD ≤21.2% were defined as having “Dry” lungs; the cut-off was determined in a control cohort as the upper limit of normal LWD (two SD above the mean).


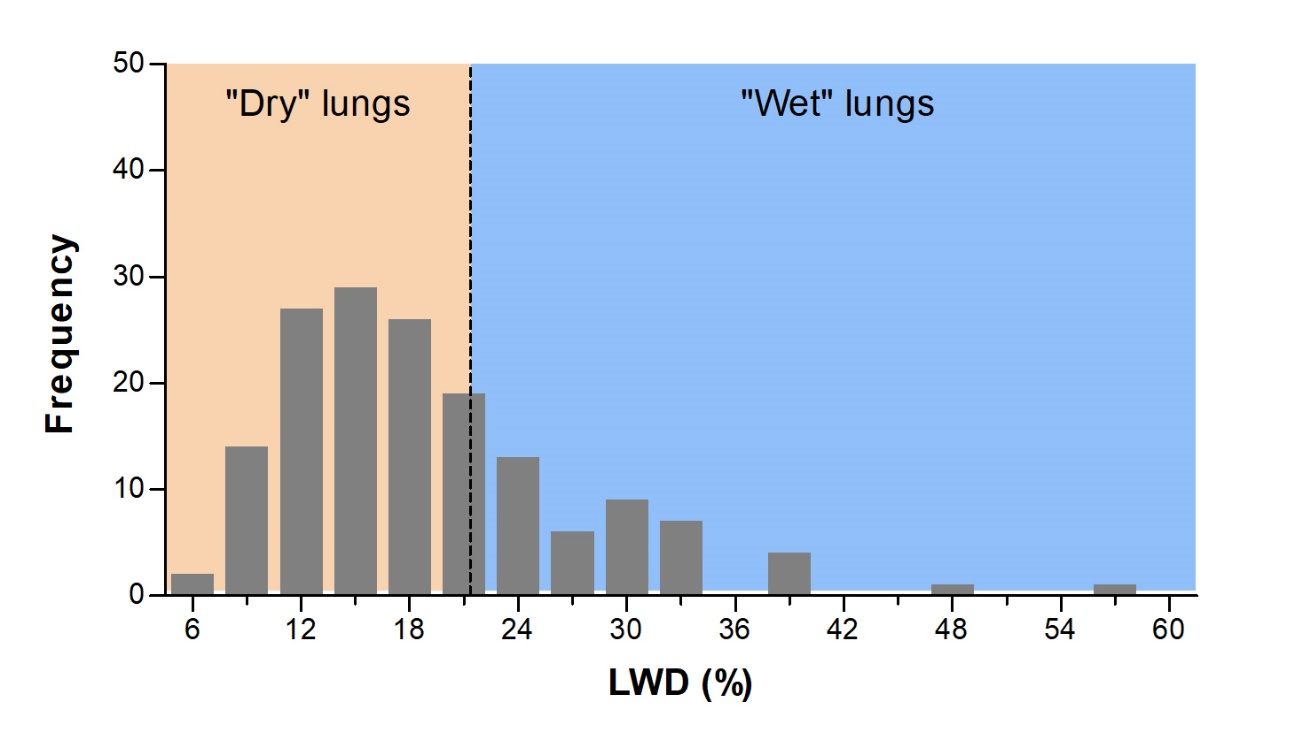


LWD = Lung Water Density.

**Supplementary Figure 3** – Correlation between LWD and NT-proBNP or LVEF.

LVEF = Left Ventricular Ejection Fraction; LWD = Lung Water Density;

**
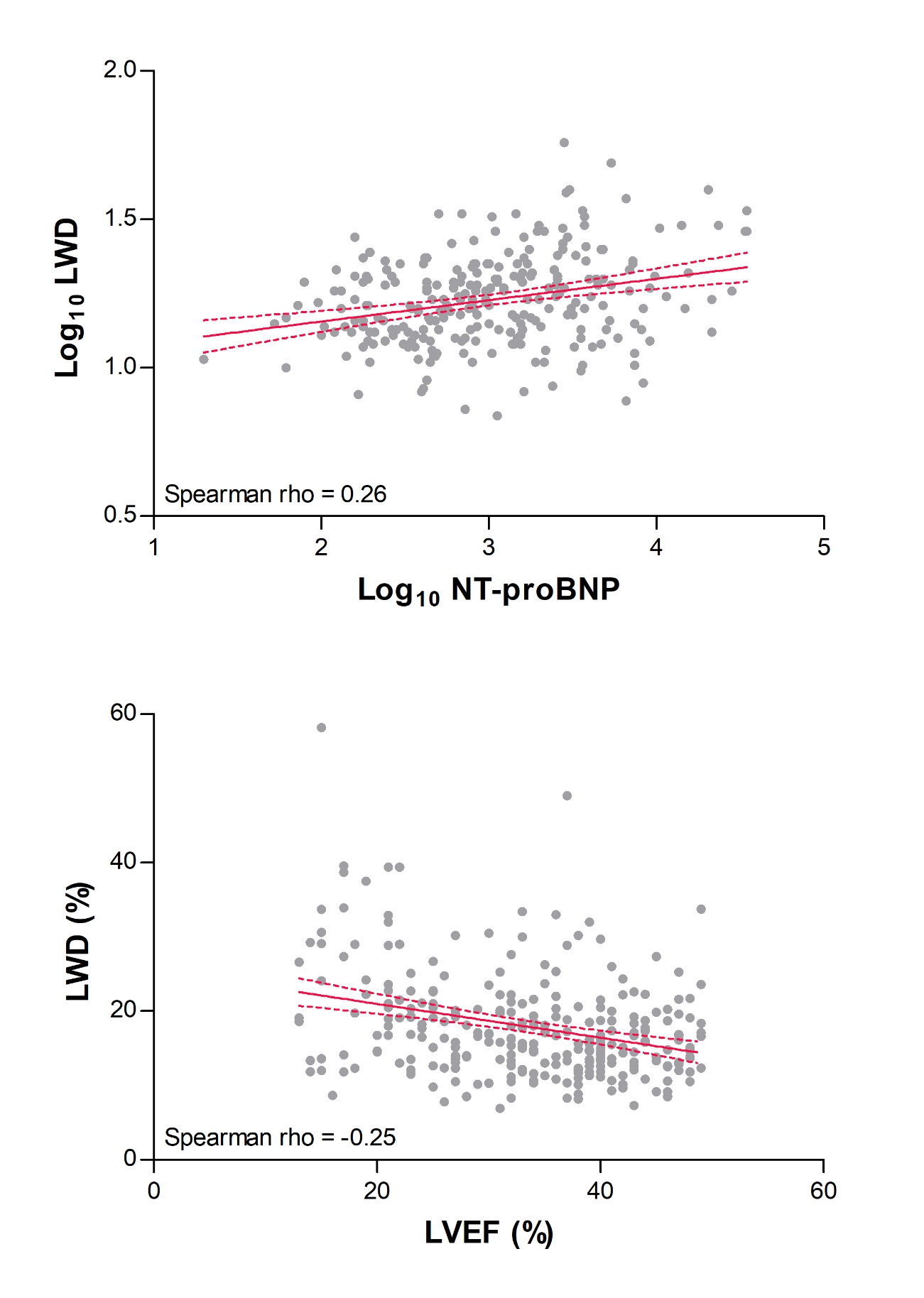
**

**Supplementary Figure 4** – Proportion of patients with normal vs*.* increased LWD across subgroups of patients with worsening NYHA (I, II, III and IV), LVEF (>40%, 35-40%, 25-35% and <25%) and NT-proBNP (increasing quartiles along the x axis);


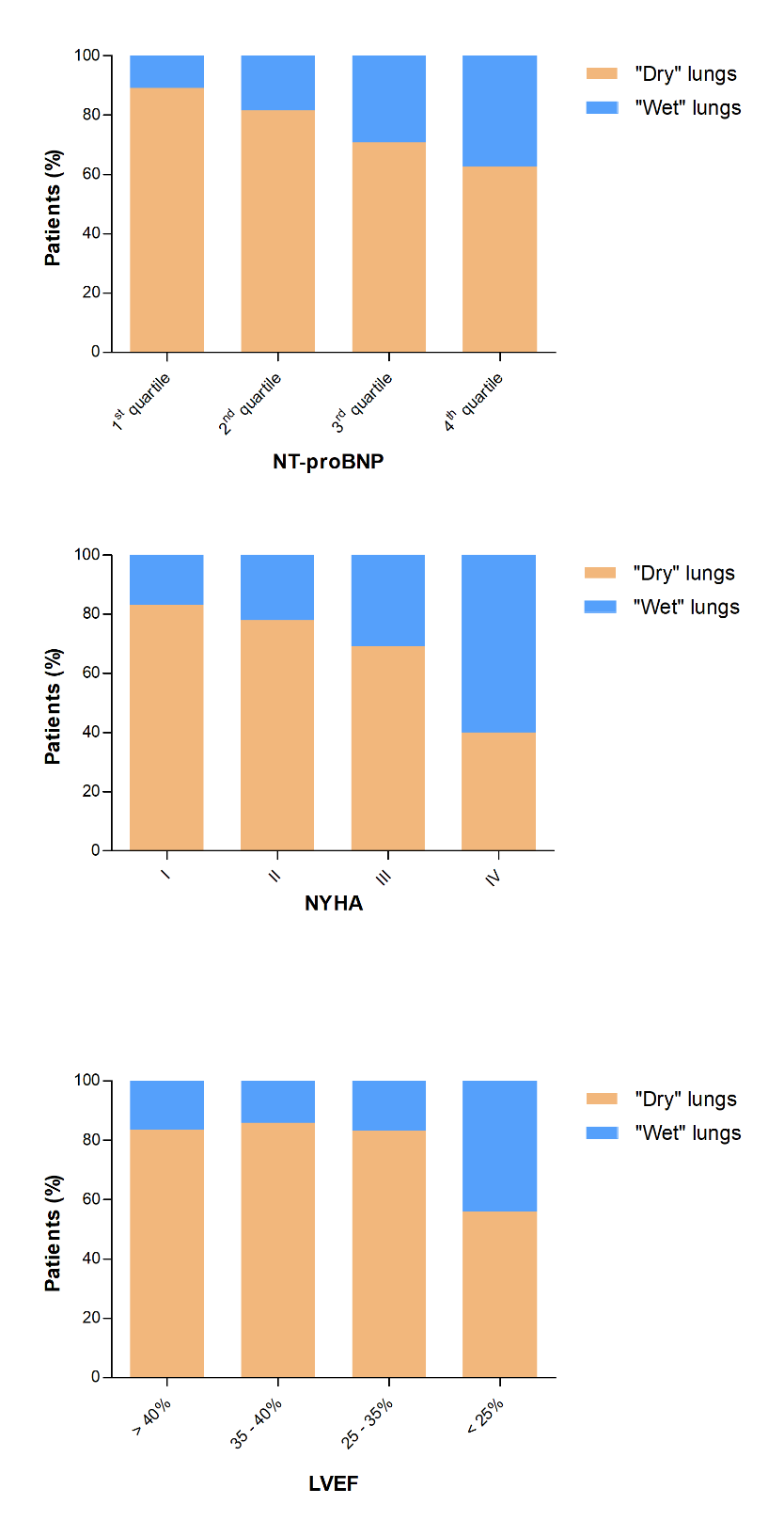


LVEF = Left Ventricular Ejection Fraction; LWD = Lung Water Density; NYHA = New York Heart Association;

**Supplementary Table 1**: Univariate and multivariate Cox Regression Model for time to first hospitalization (adjusted for competing risks).

| Variables | Univariate analysis | | | Multivariate analyses | | |
| --- | --- | --- | --- | --- | --- | --- |
|  | HR | 95% CI | p-value | HR | 95% CI | p-value |
| NYHA functional class^†^ | 4.416 | 2.363-8.253 | <0.001 | 2.585 | 1.211-5.518 | 0.014 |
| NT-proBNP, per 100pg/mL | 1.006 | 1.003-1.010 | <0.001 | 1.000 | 0.950-1.056 | 0.915 |
| Serum creatinine, per 1mg/dL | 2.125 | 1.372-3.291 | 0.001 | 1.613 | 0.818- 3.181 | 0.167 |
| LVEF, per 1% | 0.923 | 0.892-0.954 | <0.001 | 0.949 | 0.910-0.990 | 0.016 |
| LWD, per 1% | 1.107 | 1.067-1.149 | <0.001 | 1.063 | 1.007-1.122 | 0.026 |

All variables (except NYHA functional class) were assessed as continuous variables; ^†^ - NYHA III-IV versus I-II; CI = Confidence Interval at 95%; HR = Hazard Ratio; LVEF = Left Ventricular Ejection Fraction; LWD = Lung Water Density (%).
